# The mediating role of parental early maladaptive schemas in the relationship between parental self-efficacy and social interest in students with learning disabilities

**DOI:** 10.3389/fpsyt.2026.1762731

**Published:** 2026-04-10

**Authors:** Jianbin Chen, Fangfang Wu, Yinpin Huang, Deng Pan

**Affiliations:** 1 School of Literature and Cultural Communication, Tianshui Normal University, Tianshui, Gansu, China; 2 School of Accounting, Xianning Vocational Technology College, Xianning, Hubei, China; 3 School of Foreign Languages, Jingchu University of Technology, Jingmen, Hubei, China; 4 School of Foreign Languages, Hubei University of Science and Technology, Xianning, Hubei, China

**Keywords:** early maladaptive schemas, learning disabilities, parents, self-efficacy, social interest, students

## Abstract

**Introduction:**

The present work is investigating the mediating role of parental early maladaptive schemas in the relationship between parental self-efficacy and social interest among students with learning disabilities.

**Methods:**

This descriptive-analytical study employed a correlational design within a structural equation modelling framework. The population of the current work consisted of all elementary school students with a formal diagnosis of Specific Learning Disorder (SLD) in Hubei, China, during the first quarter of the 2024–2025 academic year. A sample of 200 students with specific learning disabilities was selected using a convenience sampling method. The Young Schema Questionnaire-Short Form (SQ-SF), Bandura’s Parental Self-Efficacy Scale (1994), and Alizadeh and colleagues’ Social Interest Questionnaire (2013) were used. Analysis of data was conducted using path-analysis through structural equation modelling with AMOS and SPSS-24 software.

**Results and discussion:**

The findings revealed a significant positive relationship between parental self-efficacy and students’ social interest. Additionally, there was a significant negative relationship between early maladaptive schemas and both parental self-efficacy and students’ social interest. Furthermore, the results indicated that the relationship between parental self-efficacy and students’ social interest is significant. However, when the mediator variable (early maladaptive schemas) was introduced in to the model, the path coefficient decreased. So, it was concluded that the mediating role of early maladaptive schemas in the relationship between parental self-efficacy and students’ social interest was confirmed (p ≤0.05). Therefore, these findings can contribute to the development of more effective educational policies for students with learning disabilities by emphasizing the crucial role of parents in their children’s social interests and academic success.

## 1 Introduction

Based on DSM-5-TR, the diagnosis of a Specific Learning Disorder is based on persistent difficulties in learning reading, writing, and mathematics, which must persist for at least six months (1). This disorder involves partial brain dysfunction, perceptual disabilities, cerebral impairment, dyslexia, and aphasia. However, it excludes children whose learning difficulties are primarily due to other reasons, such as motor, visual, or hearing impairments, emotional disturbances, or economic or cultural disadvantages, and intellectual disabilities (2). Reports indicate a prevalence of 10 to 15% for learning disorders among elementary school students. The highest and lowest prevalence rates were initially related to mathematics disorder (9.83%) and reading disorder (4.48%), both reported to be more common in girls than boys. Furthermore, the prevalence rates for reading, writing, and mathematics problems were 28.6%, 26.5%, and 73.8%, respectively (3). Since these disorders are associated with psychological consequences, including reduced social interest, identifying related factors such as parental self-efficacy and their maladaptive schemas is of great importance.

In this regard, studies such as (4) suggested that in children with learning disorders, the component of social interest is weak, and indeed, the presence of a learning disorder can be associated with low social interest. Social interest, meaning the tendency to cooperate and a sense of belonging to the community, is considered a sign of mental health from Adler’s viewpoint. Individuals with this characteristic possess empathy, cooperation, and optimism. In contrast, a lack of social interest leads to self-centredness, feelings of loneliness, and a need for attention. Therefore, this construct plays a key role in the quality of interpersonal relationships (5).

Based on research, there is a relationship between social interest and its indicators (Such as responsibility and empathy) in students with learning disorders and parental self-regulation and self-efficacy (6). Findings indicate that parental self-efficacy is among the related and predictive factors in explaining the social interest of these students (7). For example, one study showed a significant relationship between family functioning and parental self-efficacy with adjustment and social interest. Ultimately, these two variables are capable of predicting the adjustment and social interests of students (8).

Self-efficacy is defined as an individual’s capacity to identify life stressors and subsequently apply effective coping strategies to maintain and protect health. This sense is an individual orientation towards life (9). Enhancing the sense of self-efficacy, especially in parents, can help the family understand and guide the environment towards meaningful and appropriate behaviour, thereby making the three fundamental concepts of comprehensibility, manageability, and meaningfulness of events possible for their children and those around them (10).

Studies show that parental self-efficacy plays a significant role in reducing psychological issues (11). Parents with high self-efficacy typically exhibit more self-management and self-care behaviours and show more adaptive reactions when facing stressors such as maladaptive schemas. Conversely, low self-efficacy makes individuals more vulnerable to challenges (12). Research has also confirmed an inverse relationship between high self-efficacy and psychological problems. For this reason, this construct has long been a focus for psychology and education specialists in the field of learning disorders (13).

Furthermore, among the related and determining constructs associated with parental self-efficacy, one can point to early maladaptive schemas in these individuals. Based on Young’s (14) view, early maladaptive schemas are deep or pervasive patterns or themes that develop in childhood or adolescence and persist throughout life, relating to the individual’s relationship with themselves and others in a way that either strengthens and fosters relationships or leads to damage and challenges in this area (15). The increase of these patterns is highly dysfunctional, organising individuals’ awareness about themselves and their surrounding world, and determining their identity and their perception of the world (16). Joshua et al. (17) stated that early maladaptive schemas act like a filter for confirming or validating childhood experiences, and individuals, throughout their lives and in relationships with others—particularly in parenting and marital relationships—act in ways that confirm their schemas. On the other hand, when schemas, especially those in the domain of impaired autonomy and performance, are activated, since individuals with these schemas lack sufficient capacity for independence and autonomous functioning, they face problems in self-efficacy and relationship regulation (18).

Zubenko (19), in their study, examined the relationships between parental self-efficacy and children’s interests, intentions, and achievements from a social cognitive perspective and showed that early maladaptive schemas in parents have a significant relationship with the formation of specific behavioural styles in children, leading to decreased interest and academic achievement in their children. Therefore, early maladaptive schemas in parents reduce the sense of self-efficacy in their children and thereby play a negative role in their children’s social and academic interests and motivations.

A review of the research background revealed that although no study has directly investigated social interest in students with learning disorders, findings indicate an indirect relationship between parental self-efficacy and their maladaptive schemas with the social interest of these students. Due to interpersonal problems, these students often experience social rejection, low self-esteem, and difficulties in communication, all of which negatively impact their social interest. Therefore, based on the mentioned points and considering the research gap in investigating social interest and the relationship of psychological components of maladaptive schemas and self-efficacy in their parents with self-regulation, especially in the population of students with learning disorders, the present study was conducted to compensate for the gaps in previous research. This study is investigating the mediating role of parental early maladaptive schemas in the relationship between parental self-efficacy and social interest in students with learning disorders.

## 2 Method

A structural equation modelling (SEM) approach was applied to evaluate the proposed relationships between variables. In terms of objective, the research is classified as applied.

### 2.1 Participants and sampling

The target population for this research comprised all elementary school students with a formal diagnosis of Specific Learning Disorder (SLD) in Hubei, China, during the first quarter of the 2024–2025 academic year.

A convenience sampling method was employed to recruit participants. The sample size was determined using two complementary methods to ensure robustness. First, Kline’s (20) guideline for Structural Equation Modelling (SEM), which recommends 10–20 participants per observed variable, was considered. Second, an *a priori* power analysis was performed using specialised software (21). With parameters including an anticipated effect size of 0.19, a statistical power of 0.95, and an alpha level of 0.05, the analysis recommended a minimum sample size of 195. To account for potential attrition and incomplete responses, a final sample of 200 student-parent dyads was recruited.

Prior to data collection, the purpose and procedures of the study were thoroughly explained to both parents and their children. Participation in this study involved no financial cost for the participants, and they were free to withdraw from the study at any time if they wished. In the event of any specific issues related to the research, the participants were duly informed.

Inclusion criteria for students were (a) active enrolment in an elementary school, (b) a formal diagnosis of SLD confirmed by educational or clinical records, and (c) verification of normal intellectual capacity. Exclusion criteria were (a) the presence of comorbid sensory or motor impairments, (b) a diagnosis of any other major psychological or neurodevelopmental disorders, or (c) a diagnosis of intellectual disability.

### 2.2 Measures

#### 2.2.1 Demographic information sheet

A researcher-developed questionnaire was used to gather data on participant characteristics, such as the age and gender of children, as well as parental education level, socioeconomic status, and psychological history.

#### 2.2.2 Young Schema Questionnaire-Short Form

Early Maladaptive Schemas (EMSs) were assessed using the 75-item YSQ-SF (22). This self-report instrument measures 15 maladaptive schemas across domains such as Disconnection/Rejection (e.g., Emotional Deprivation, Social Isolation), Impaired Autonomy (e.g., Dependence, Vulnerability to Harm), and Other-Directedness (e.g., Subjugation, Self-Sacrifice). Parents rated items on a 6-point Likert scale ranging from 1 (*Completely untrue of me*) to 6 (*Describes me perfectly*). Schema scores are calculated by averaging the five items for each subscale, with higher scores indicating stronger endorsement of that schema. The scale has demonstrated strong psychometric properties. Bachicha (23) reported excellent internal consistency (α = 0.95) and good test-retest reliability (r = 0.81). Welburn et al. (24) further confirmed its validity and reliability across different groups.

#### 2.2.3 Bandura’s Parental Self-Efficacy Scale

Parental self-efficacy was measured using the 55-item scale developed by Bandura (25). Parents indicate their confidence in managing various parenting challenges on a 4-point Likert scale. The instrument yields a total score (ranging from 0 to 100) and encompasses four factors: coping with school-related issues, managing difficult circumstances, providing care, and exercising control/supervision. Bachicha (23) reported high reliability for the total scale (α = 0.90) and its subscales (α ranging from 0.81 to 0.91).

#### 2.2.4 Crandall Social Interest Scale

Social interest in students was evaluated using the 24-item CSIS (26). This scale, grounded in Adlerian theory, assesses feelings of belonging, cooperation, and responsibility towards society (e.g., “I feel a sense of equality with all people”). Students respond on a 5-point Likert scale from 1 (*Strongly Disagree*) to 5 (*Strongly Agree*), with some items reverse-scored. Total scores range from 24 to 120, with higher scores reflecting greater social interest. The scale has shown strong internal consistency in previous research, with Simon (27) reporting a Cronbach’s alpha of 0.92 for the total score.

### 2.3 Procedure

Data collection was conducted in two phases. First, a comprehensive review of the theoretical and empirical literature was conducted to establish the foundational model. Subsequently, the field study was implemented.

After obtaining necessary permissions from relevant educational authorities in Hubei, China, eligible student-parent dyads were identified through collaboration with local schools and educational support centres. Potential participants were provided with a detailed explanation of the study’s purpose, procedures, and the voluntary nature of their participation.

Prior to data collection, the purpose and procedures of the study were thoroughly explained to both parents and their children. All participants were assured of the confidentiality of their responses and their right to withdraw from the study at any time without any penalty or negative consequences. Participation in this study involved no financial cost for the participants.

The research questionnaires were administered electronically via a secure online platform to ensure accessibility and efficient data management. Parents completed the Young Schema Questionnaire-Short Form (YSQ-SF) and Bandura’s Parental Self-Efficacy Scale, while students completed the Crandall Social Interest Scale (CSIS). The online platform was designed to be user-friendly and allowed participants to complete the questionnaires at their convenience within a specified timeframe.

Upon completion of data collection, and upon request, a summary of the findings was provided to participants. Furthermore, if any assessment indicated significant psychological concerns, appropriate guidance and referral information were offered to the relevant families. All data were stored securely and accessed only by the research team for analytical purposes.

#### 2.3.1 Data analysis plan

Data analysis was performed using SPSS version 24 and AMOS version 24. The process involved two main stages:

#### 2.3.2 Descriptive statistics

The data were first screened for missing values and outliers. Means, standard deviations, and frequency distributions were calculated for all study variables to describe the sample.

#### 2.3.3 Inferential statistics

The hypothesised structural equation model was tested. The analysis followed a two-step procedure recommended for SEM:

#### 2.3.4 Measurement model

Confirmatory Factor Analysis (CFA) was conducted to assess the validity and reliability of the latent constructs (Social Interest, Parental Self-Efficacy, and the domains of Early Maladaptive Schemas).

#### 2.3.5 Structural model

The path coefficients between the constructs were estimated to test the direct and indirect (mediating) relationships outlined in the research hypotheses. Model fit was evaluated using standard indices such as χ²/df, CFI, TLI, and RMSEA. The significance of the mediation effects was tested using bootstrapping procedures.

## 3 Strengths and limitations of the study

### 3.1 Strengths of the study

1. Novel contribution: This research addresses an under-researched area by examining the mediating role of early maladaptive schemas in the relationship between parental self-efficacy and social interest specifically among students with learning disabilities, providing a comprehensive understanding of family processes influencing social development in this vulnerable population.
2. Robust methodological approach: The use of structural equation modelling (SEM) allowed for simultaneous examination of complex relationships among multiple variables while accounting for measurement error. Confirmatory factor analysis (CFA) ensured adequate validity and reliability of all constructs prior to path analysis.
3. Adequate sample size: Sample size determination through both (20) guideline and *a priori* power analysis (21) enhanced statistical robustness and reduced the risk of Type II error.
4. Validated instruments: All measures demonstrated strong psychometric properties, with Cronbach’s alpha coefficients exceeding 0.70 for all variables and factor loadings above 0.4 with significant critical ratios.
5. Multi-informant approach: Inclusion of both parents and children as informants reduced potential for common method bias and provided a more nuanced examination of family dynamics.
6. Clinical relevance: Focus on elementary school students with diagnosed learning disabilities addresses an important population vulnerable to social difficulties, with findings offering practical implications for intervention development.

### 3.2 Limitations of the study

1. Sampling constraints: The use of convenience sampling from a single geographic region (Hubei, China) limits generalisability to broader populations. Findings may not extend to other regions within China or to international contexts.
2. Cross-sectional design: The cross-sectional design precludes conclusions about causal relationships. Longitudinal research is necessary to establish temporal precedence and examine how these relationships evolve over time.
3. Self-report measures: Reliance on self-report introduces potential response biases, including social desirability bias and common method variance. Future research should incorporate observational methods or teacher reports.
4. Modest effect sizes: All effect sizes were small to modest (β ranging from 0.133 to 0.240), indicating that parental factors explain only limited variance in children’s social interest. Other important variables such as peer relationships, teacher influences, and school environment warrant investigation.
5. Population specificity: The sample exclusively comprised elementary school students with diagnosed learning disabilities, limiting applicability to other age groups or typically developing populations.
6. Unexamined moderators: Potential differences based on child gender, type of learning disability, or socioeconomic status were not examined. Future studies with larger samples should explore these moderating effects.
7. Limited parental variables: While focussing on early maladaptive schemas, other parental psychological processes such as self-esteem, hopelessness, and emotion regulation strategies were not included. Recent research highlights the significance of these factors in families of children with special needs (28).
8. Cultural specificity: The study was conducted within a specific cultural context (China). Findings may not translate directly to Western or other non-Asian cultural settings where parenting practices and social expectations differ. Cross-cultural comparative research is needed.

## 4 Results

This study investigated 200 parents of students with specific learning disorders (111 fathers and 89 mothers) with an age range of 25 to 55 years (Mean = 36.93, SD = 12.22). The student participants included 98 boys (49%) and 102 girls (51%) aged 7 to 12 years (Mean = 9.21, SD = 3.14).

The distribution of students across grade levels was as follows: first grade (22.5%), second grade (17.5%), third grade (20%), fourth grade (25%), and fifth grade (15%). Regarding parental education level, 3.5% had less than a high school diploma, 22.5% held a high school diploma or associate degree, 50% held a bachelor’s degree, 15% held a master’s degree, and 6% held a doctoral degree.

The socio-economic status of the sample indicated that 50% were in the middle class, 27.5% in the high class, and 22.5% in the low class. Concerning the type of learning disorder, 29.5% had a reading disorder, 40% a writing disorder, 17.5% a mathematics disorder, and 13% a mixed disorder. The mean intelligence quotient (IQ) of the students was 89.80 (SD = 3.013), ranging from 80 to 98.

Prior to testing the main research study hypotheses, the normality of the distribution for the studied variables was assessed to determine whether parametric or non-parametric methods should be used for hypothesis testing. “A normally distributed dataset exhibits symmetrical dispersion around the mean, producing the characteristic bell-shaped probability curve. In contrast, departures from normality are observed when the distribution demonstrates asymmetry, resulting in skewness either to the left or right of the mean. Assessment of normality was conducted through examination of skewness and kurtosis indices. Consistent with established methodological conventions, the data were considered to satisfy the normality assumption when the absolute values of skewness and kurtosis remained within the ±2 threshold as per **Table 1**.

**Table 1:** Descriptive statistics for the research variables (N = 200).

Variable / Subscale	Min	Max	Mean	SD
Early Maladaptive Schemas				
Emotional Deprivation	1	7	6.76	1.43
Abandonment	1	6	5.62	1.06
Social Isolation	2	12	11.25	1.72
Defectiveness/Shame	1	11	9.81	1.2
Failure	2	13	10.07	1.42
Dependence	1	5	4.67	1.14
Vulnerability	1	10	8.33	1.04
Enmeshment	1	8	7.2	1.31
Subjugation	3	13	8.33	11.12
Self-Sacrifice	2	11	8.95	1.31
Emotional Inhibition	1	7	2.66	11.83
Unrelenting Standards	1	9	6.21	1.14
Entitlement	3	11	10.97	1.36
Insufficient Self-Control	1	12	7.63	0.98
Approval-Seeking	3	13	9.15	0.53
Total Schema Score	83	441	241.08	66.65
Parental Self-Efficacy				
Coping with School Issues	12	25	7.24	1.87
Coping with Difficult Situations	18	41	18.57	2.01
Providing Care	9	35	16.41	2.25
Control and Supervision	11	40	17.06	2.01
Total Self-Efficacy Score	26	81	56.11	12.14
Social Interest				
Responsibility and Task Performance	49	99	87.33	11.7
Connection with People and Empathy	33	94	80.05	12.03
Courage and Confidence	17	54	41.11	6.78
Sense of Equality vs. Inferiority	16	72	48.11	6.19
Total Social Interest Score	146	281	238.8	26.06

Min, Minimum; Max, Maximum; Std, Standard Deviation.

The results showed that the skewness and kurtosis values for all variables were between -2 and +2. It can be inferred that no normal distribution violation is observed in the data. So, overall, and based on the conducted assessments, it can be said that the variable distribution in the table above is normal, or close to normal.

As demonstrated in **Table 2**, Pearson correlation analysis revealed statistically significant relationships among all primary research variables. The analysis yielded a significant negative correlation between early maladaptive schemas and parental self-efficacy (R = -0.20, p <.01), showing that higher levels of maladaptive schemas were related to lower levels of self-efficacy in parents. Furthermore, a significant positive correlation was observed between parental self-efficacy and children’s social interest (R = 0.22, p <.01), suggesting that parents with higher self-efficacy tended to have children with greater social interest. Additionally, early maladaptive schemas revealed a significant negative correlation with social interest (R = -0.13, p <.05), demonstrating that higher schema scores were related to lower levels of social interest in children with learning disorders. These correlational patterns provide preliminary support for the proposed relationships in our theoretical model and justify further investigation through structural equation modelling to examine the potential mediating relationships.

**Table 2:** Pearson correlation coefficients among research variables.

Variables		Early maladaptive schemas	Self-efficacy	Social interest
Early Maladaptive Schemas	Correlation Coefficient	1		
	Significance Level	0.001		
Self-Efficacy	Correlation Coefficient	-0.201**	1	
	Significance Level	0.002	0.001	
Social Interest	Correlation Coefficient	-0.133*	0.222**	1
	Significance Level	0.042	0.028	0.035

*p<0.05, **p<0.01.

### 4.1 Confirmatory factor analysis of research variables

**Figure 1:**
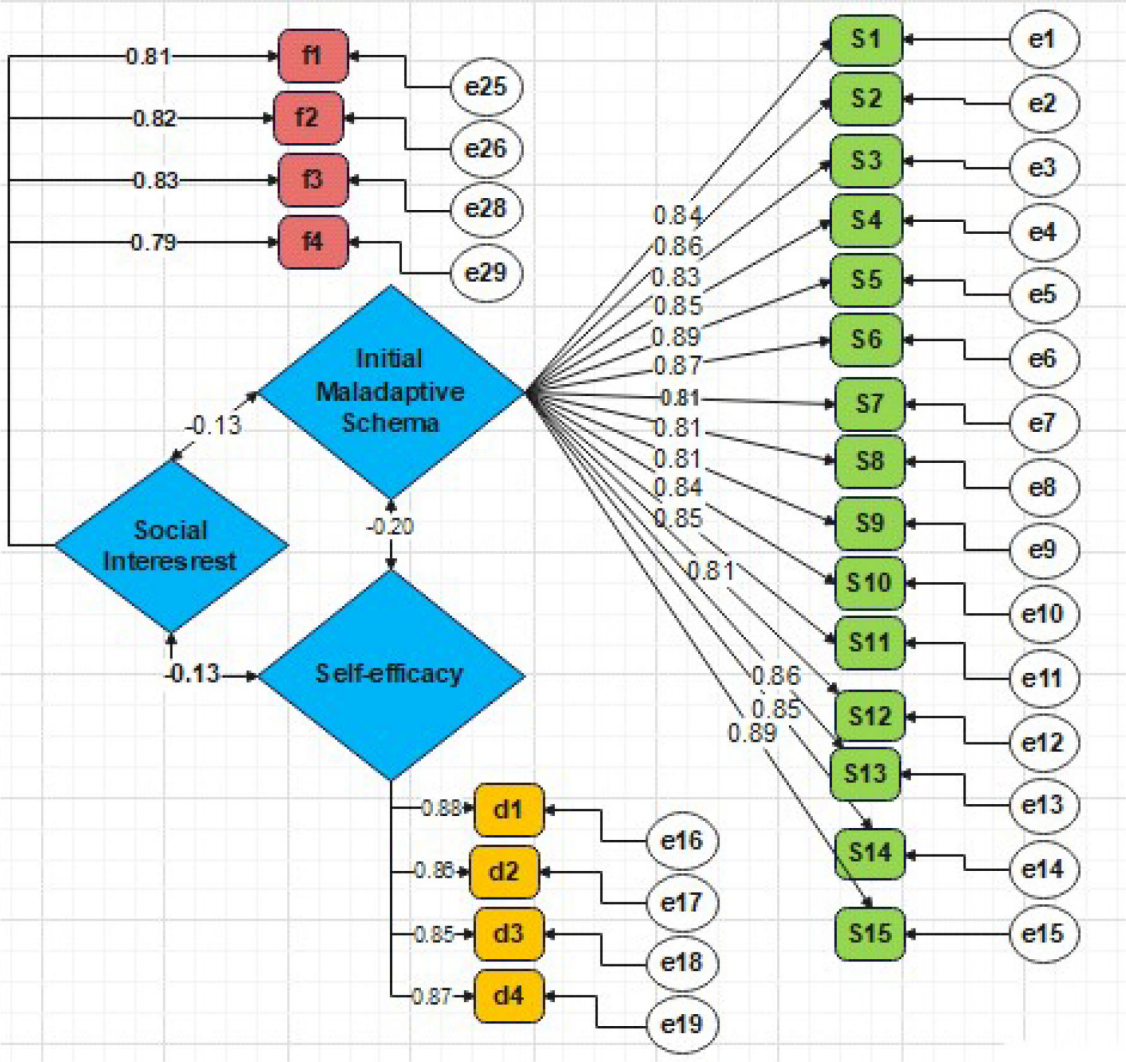
Standardised factor loading coefficients of the 1^st^ order confirmatory factor analysis (CFA) model.

First, to proceed to structural equation modelling, the research instruments must be subjected to confirmatory factor analysis to calculate construct validity. In this section of the research, using 1^st^-order confirmatory factor analysis (CFA) as per **Figure 1**, the validity of the questionnaire items related to the research variables has been assessed.

### 4.2 Interpretation of standardised coefficients in the measurement model

The standardised coefficients from the measurement model indicate whether significant correlations exist between the latent variables and their corresponding indicators. These standardised coefficients represent the path coefficients or standardised factor loadings between the factors and their markers. For validity to be established, there must be a significant correlation between the construct and its corresponding indicators.

If the standardised factor loading is more than 0.4, it can be concluded that the respective items possess great explanatory power. The critical ratio (CR) values demonstrate the significance of each parameter. If the CR value is greater than or equal to the absolute value of 1.96, the parameters investigated in the model are considered significant.

As observed in **Table 3**, the standardised factor loadings for all items are greater than 0.4. Furthermore, since all critical ratios for the relationships between the items and their corresponding latent variables exceed 1.96, with significance levels below the 0.05 error threshold, it can be stated that the construct validity of the measurement tools for the variables is confirmed at the 0.05 significance level. Therefore, no need is felt to modify or remove any items from the model or the research questionnaires. The results of Cronbach’s alpha coefficient for the studied variables are also presented, which was more than 0.7 for all the variables.

**Table 3:** Standardised factor loadings and significance levels between questionnaire items and research variables.

Component/Indicator	Questionnaire Item	Standardised Coefficient	Critical Ratio (C.R.)	Significance Level	Cronbach's Alpha	Result
Early Maladaptive Schemas					0.973	
	s1	0.835	-	-		Acceptable
	s2	0.857	19.073	***		Acceptable
	s3	0.832	18.172	***		Acceptable
	s4	0.853	18.927	***		Acceptable
	s5	0.888	20.308	***		Acceptable
	s6	0.869	19.55	***		Acceptable
	s7	0.806	17.266	***		Acceptable
	s8	0.805	17.237	***		Acceptable
	s9	0.811	17.423	***		Acceptable
	s10	0.818	18.371	***		Acceptable
	s11	0.848	18.74	***		Acceptable
	s12	0.815	17.564	***		Acceptable
	s13	0.864	19.371	***		Acceptable
	s14	0.852	18.905	***		Acceptable
	s15	0.804	17.19	***		Acceptable
Self-Efficacy					0.966	
	d1	0.876	-	-		Acceptable
	d2	0.859	20.866	***		Acceptable
	d3	0.862	21.023	***		Acceptable
	d4	0.845	20.188	***		Acceptable
Social Interest					0.958	
	f1	0.807	-	-		Acceptable
	f2	0.823	16.785	***		Acceptable
	f3	0.814	16.521	***		Acceptable
	f4	0.788	15.784	***	Cronbach's Alpha	Acceptable

***p<0.001.

### 4.3 Model fit indices

In order to validate of the model and results, it is needed to get the model fit indices in an acceptable range. The indices used, along with their values, are presented in the table below. As observed in **Table 4**, in the research model, the (χ²/df) is 1.204, which is lower than 3. Furthermore, the RMSEA is 0.026, less than the 0.08 threshold. Additionally, the Comparative Fit Index (CFI), the Incremental Fit Index (IFI), and the Goodness of Fit Index (GFI) are 0.98, 0.98, and 0.87, respectively, all falling within a highly desirable range. Therefore, the model demonstrates a good fit and is confirmed.

**Table 4:** Fit indices of the factor analysis model.

Fit Index	Symbol	Standard value	Estimated value
Chi-square/Degrees of Freedom	(χ²/df)	< 3	1.202
Root Mean Square Error of Approximation	(RMSEA)	< 0.08	0.026
Comparative Fit Index	(CFI)	> 0.90	0.98
Incremental Fit Index	(IFI)	> 0.90	0.98
Goodness of Fit Index	(GFI)	> 0.80	0.87

The confirmatory factor analysis demonstrated that the factor loadings between the questionnaire items and their corresponding latent variables were statistically significant, and there is no need to remove or modify any questionnaire items. Furthermore, the results of the model fit indices, as well as the Cronbach’s alpha values, are at a desirable and reliable level. Thus, based on the collected data and with 95% confidence, it can be stated that the questionnaire items indeed measure what they are intended to measure.

Subsequently, the results of the path analysis for examining the direct relationships between the research variables are presented in **Table 5**. As observed, all hypotheses concerning the direct relationships in the model are statistically significant. Accordingly, parental self-efficacy, early maladaptive schemas, and students’ social interest have significant relationships with each other.

**Table 5:** Path analysis results.

Hypothesis	Critical Ratio (C.R.)	Sig	Standardised Path Coefficient	Result
Parental Self-Efficacy → Social Interest	3.585	0	0.222	Confirmed
Parental Self-Efficacy → Early Maladaptive Schemas	-3.393	0.001	-0.201	Confirmed
Early Maladaptive Schemas → Social Interest	-2.222	0.001	-0.133	Confirmed

Based on the path analysis results, all direct relationships in the research model were significant. The findings indicated that parental self-efficacy has a direct, positive, and significant effect on students’ social interest (β = 0.222, CR = 3.585, p < 0.001). In other words, with a one standard deviation increases in parental self-efficacy, students’ social interest increases by 0.222 standard deviations.

Furthermore, parents’ early maladaptive schemas showed a direct, negative, and significant relationship with parental self-efficacy (β = -0.201, CR = -3.393, p < 0.001). This means that a one-standard-deviation increase in maladaptive schemas leads to a 0.201 unit decrease in parental self-efficacy.

Additionally, a direct and inverse relationship was observed between parents’ early maladaptive schemas and students’ social interest (β = -0.133, CR = -2.222, p < 0.001). Accordingly, a one standard deviation increases in parental maladaptive schemas results in a 0.133 unit decrease in students’ social interest. All these relationships were confirmed at a 95% confidence level.

The Baron and Kenny exam was applied for assessing the effect of early maladaptive schemas between the predictor variable (parental self-efficacy) and the criterion variable (social interest). In this section, aside from the direct association between parental self-efficacy and students’ social interest, its indirect relationship through the mediating variable of early maladaptive schemas can also be obtained. For this purpose, the Baron and Kenny method was used. To establish the following conditions, it is necessary:

The 1^st^ requirement is that the independent variable must show a significant association with the dependent variable.

The 2^nd^ requirement is that the relationship between the independent variable and the mediating variable must be shown to be significant.

The 3^rd^ requirement is the confirmation of the significant relationship between the mediating and dependent variables.

The 4^th^ requirement is that once the mediating variable is involved in the regression equations, the independent variable no longer significantly predicts the dependent variable, or this relationship decreases (at least 0.10) but remains significant (in which case the mediating variable plays a partial role).

According to the results provided in **Table 6**, the mediation analysis conducted via the Baron and Kenny (29) method confirms that early maladaptive schemas play a partial mediating role in the relationship between parental self-efficacy and students’ social interest.

**Table 6:** The relationship between parental self-efficacy and students' social interest, considering the role of early maladaptive schemas as mediators.

Steps of the Baron and Kenny Test	Path	Path Coefficient	Critical Value	Sig
Model 2	Parental Self-Efficacy → Social Interest	0.240	3.848	0.001
(Model 3)	Parental Self-Efficacy → Early Maladaptive Schemas	-0.201	-3.393	0.001
	Early Maladaptive Schemas → Social Interest	-0.165	-2.712	0.007
	Parental Self-Efficacy → Social Interest	0.222	3.585	0.001

**Figure 2:**
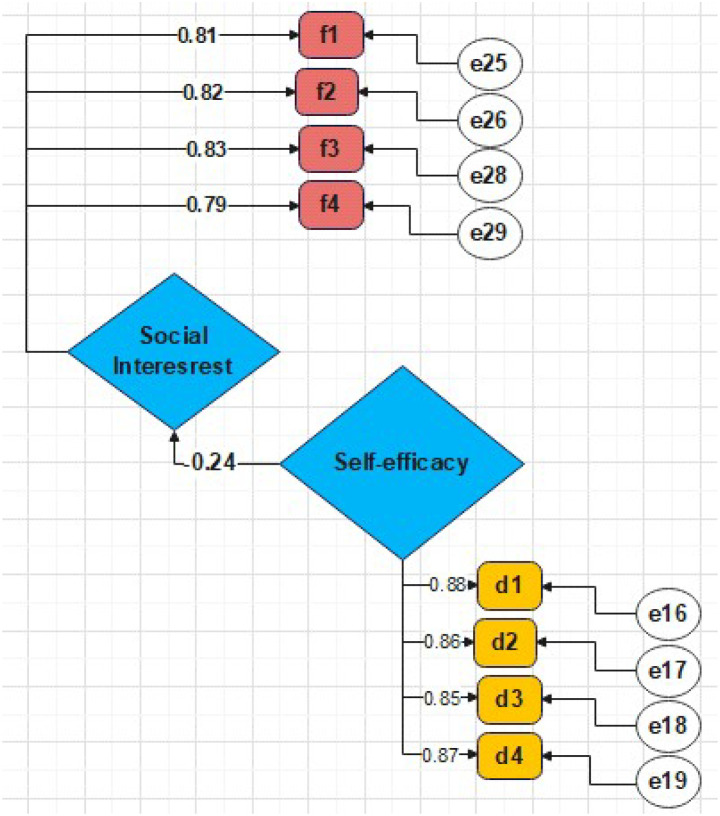
Standardised path coefficients between parental self-efficacy and students’ social interest without the intervention of the mediating variable of early maladaptive schemas (Condition 1).

The analysis proceeded in two key stages. First, the direct effect of parental self-efficacy on social interest was established without the mediator (**Figure 2**), showing a significant path coefficient (β = 0.240, p < 0.001). This satisfied the first condition for mediation.

**Figure 3:**
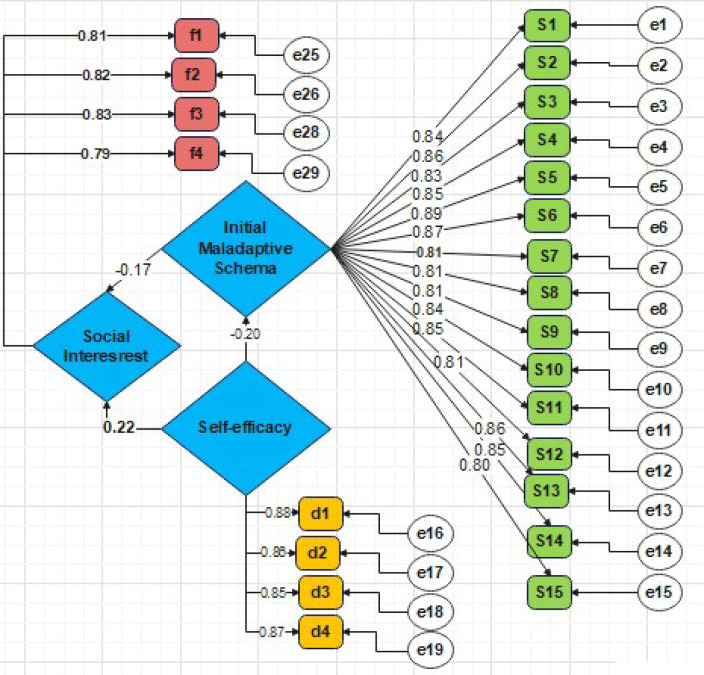
Research model with standardised path coefficients.

In the second stage, the mediator was introduced (**Figure 3**). The paths from parental self-efficacy to early maladaptive schemas (β = -0.201, p < 0.001) and from early maladaptive schemas to social interest (β = -0.165, p < 0.01) were both significant, fulfilling the second and third conditions. Furthermore, upon the inclusion of the mediator, the direct path coefficient from parental self-efficacy to social interest decreased from 0.240 to 0.222 but remained statistically significant (p < 0.001).

This reduction in the direct path coefficient, while it remains significant, indicates a pattern of partial mediation. This finding suggests that parental self-efficacy influences students’ social interest through two distinct pathways: a direct positive effect and an indirect effect that operates by reducing the early maladaptive schemas presence in parents, which in turn is related to more social interest in their children.

## 5 Discussion

The present study examined the relationships between parental self-efficacy, early maladaptive schemas, and social interest in students with learning disabilities.

The results demonstrated a positive relationship between parental self-efficacy and students’ social interest (β = 0.222, p < 0.001). While this effect is statistically significant, the magnitude indicates that parental self-efficacy explains approximately 5% of the variance in students’ social interest. This modest effect size suggests that although parental self-efficacy contributes positively to children’s social development, it is one of multiple factors influencing social interest in this population. From a practical perspective, this finding implies that interventions aimed at enhancing parental self-efficacy may yield small but meaningful improvements in students’ social interest. For children with learning disabilities who often experience social difficulties, even modest gains in social interest—such as increased willingness to cooperate with peers or a slightly greater sense of belonging—can have meaningful implications for their daily functioning and quality of life. This finding aligns with Bandura’s social learning theory, which posits that parents with high self-efficacy act as effective models who, through modelling, positive reinforcement, and creating constructive expectations, provide the grounds for developing social skills in their children (30). Additionally, attachment theory suggests that parental self-efficacy leads to secure attachment in children, which in turn reinforces the sense of security and trust necessary for healthy social interactions (31). From Adler’s theoretical perspective, social interest, as the cornerstone of mental health, is nurtured in families where parents have high efficacy, as such parents provide a supportive, structured, and meaningful environment that facilitates comprehensibility and manageability of events for their children (32). Recent research has further emphasised that parental psychological resources, including self-efficacy and self-esteem, are significantly associated with children’s socio-emotional development, with parents of children with special needs demonstrating lower self-esteem and higher hopelessness levels that can indirectly affect child outcomes (28). Moreover, the intergenerational transmission of parental psychological distress through emotion socialisation processes has been identified as a critical pathway influencing children’s emotional regulation and social competence (33).

The inverse relationship between early maladaptive schemas and parental self-efficacy (β = -0.201, p < 0.001) also represents a small effect size. This suggests that while maladaptive cognitive patterns in parents are associated with reduced parenting confidence, they are not the sole determinant. The clinical significance of this finding lies in its implication that schema- focussed interventions could potentially contribute to strengthening parental self-efficacy, though such effects would likely be modest and should be combined with other supportive strategies. Based on Young’s theoretical framework, these schemas, as maladaptive cognitive patterns formed from childhood, distort parents’ perceptions of themselves and their abilities. Specifically, schemas in the domain of impaired autonomy and performance (such as Dependence/Incompetence) directly weaken parents’ beliefs about their competence in fulfilling the parenting role (13). These schemas, through cognitive mechanisms like perceptual distortions and selective attention to negative experiences, cause parents to underestimate their abilities even when facing normal parenting situations (15). Furthermore, these schemas, by influencing parenting styles and creating dysfunctional interaction cycles, gradually erode parents’ sense of competence and reduce their self-efficacy (34). This pattern is consistent with research demonstrating that parents’ psychological characteristics, including self-esteem and hopelessness, are significantly intertwined with their capacity to effectively support their children’s development, particularly in families raising children with special needs (28). Family communication patterns and parental responses to children’s emotions have also been shown to mediate the relationship between parental psychological functioning and children’s social self-efficacy (35).

Similarly, the negative relationship between early maladaptive schemas and students’ social interest (β = -0.133, p < 0.05) represents a small effect, indicating that parental schemas account for a limited portion of the variance in children’s social outcomes. This modest effect is theoretically meaningful, as it suggests that parental cognitive patterns exert an indirect and gradual influence on children’s social development through complex family processes. In practical terms, this finding highlight that addressing parental schemas alone may produce limited improvements in children’s social interest, and comprehensive interventions targeting multiple levels of the family system are likely necessary. These schemas affect children’s social development through several mechanisms: they distort the quality of parent-child interactions and pave the way for the formation of insecure attachment in children (36); parents with these schemas often lack the necessary communication skills for modelling healthy social interactions and may unconsciously reinforce avoidant or aggressive behaviours in their children; and these schemas, by creating a negative emotional atmosphere in the family, weaken the fundamental sense of belonging and trust required for children to explore social environments. Schemas in the Disconnection and Rejection domain are particularly effective in reducing students’ tendency to form social bonds (37, 38). These findings align with recent evidence that parental trauma-related distress and maladaptive emotion socialisation behaviours can significantly undermine children’s emotional regulation capacities and social competence (33). Furthermore, research on rewarding and punitive parenting practices has demonstrated that parental disciplinary approaches are differentially associated with children’s self-regulation and social competence, highlighting the importance of positive parenting strategies in fostering adaptive social outcomes (39).

The mediation analysis revealed that early maladaptive schemas partially mediated the relationship between parental self-efficacy and social interest, with the direct effect reducing from β = 0.240 to β = 0.222 upon inclusion of the mediator. The small magnitude of this indirect effect indicates that the mediating role of maladaptive schemas, while statistically significant, is relatively modest. This suggests that parental self-efficacy influences children’s social interest primarily through direct pathways—such as modelling and positive reinforcement—rather than primarily through the mechanism of maladaptive schemas. From a clinical perspective, this implies that interventions focussed directly on enhancing parental self-efficacy may be more efficient than those targeting schema modification alone, though combining both approaches could offer additional benefits. Based on Young’s theoretical framework, maladaptive schemas act as perceptual filters, limiting parents’ ability to translate positive beliefs about parenting competencies into effective supportive behaviours (40). Specifically, schemas in the Impaired Autonomy domain and the Disconnection and Rejection domain, by creating cognitive distortions in understanding children’s social needs and reducing the effectiveness of parenting strategies, disrupt the pathway through which self-efficacy influences children’s social development (41). This mediating role is consistent with broader research indicating that parental psychological processes, including self-esteem and cognitive-emotional structures, serve as important mechanisms through which family factors influence child outcomes (28).

The small to modest effect sizes observed throughout this study are consistent with the complex, multi-determined nature of social development in children with learning disabilities. Social interest is shaped by numerous factors, including peer relationships, school environment, individual temperament, and broader cultural contexts, in addition to parental characteristics. Therefore, expecting large effects from any single parental variable would be unrealistic. The practical significance of these findings should be understood within this broader context: even small improvements in parental self-efficacy and reductions in maladaptive schemas may contribute incrementally to better social outcomes for these children over time. Recent longitudinal research has similarly demonstrated that parenting stress and family conflict, while showing modest direct effects, accumulate over time to significantly predict children’s behavioural problems, particularly when the quality of parent-child interactions is compromised (42).

These findings have several implications for practice. For educators and clinicians working with families of children with learning disabilities, interventions that enhance parental self-efficacy—such as skills training, positive feedback, and supportive counselling—may yield small but meaningful improvements in children’s social interest. Additionally, incorporating schema-focussed components into parenting programs may provide modest additional benefits. However, given the small effect sizes, such interventions should be viewed as one component of comprehensive support plans that also address children’s direct social skills training, peer relationships, and school environment. Given that parents of children with special needs often experience elevated hopelessness and reduced self-esteem (28), interventions should also target these broader psychological resources to optimise both parental well-being and child outcomes.

In conclusion, while the relationships identified in this study are statistically significant, their modest magnitude reminds us that parental factors, though important, are part of a larger network of influences on social interest in students with learning disabilities. A balanced interpretation acknowledging both the meaningful contributions of parental variables and the need for multi-level interventions provides the most accurate and useful understanding for both researchers and practitioners.

Despite limitations, including the focus on elementary school students in Hubei, the use of convenience sampling, and the modest sample size which affects the generalisability of the results, this study provides a valuable foundation for future research. It is recommended that future studies employ longitudinal designs, random sampling, and larger sample sizes to investigate causal relationships among variables in more diverse populations.

Regarding practical implications for school settings, parental self-efficacy programs could be implemented through the following concrete strategies:

1. Skill-based parenting workshops: Schools can organise regular workshops where parents learn and practise specific strategies for supporting their children’s learning and social development, such as positive reinforcement techniques, effective communication skills, and structured homework assistance methods.
2. Peer support groups: Establishing parent support groups within schools where parents of children with learning disabilities can share experiences, exchange successful strategies, and receive emotional support from others facing similar challenges.
3. Individual coaching sessions: Providing parents with access to school counsellors or educational psychologists for personalised guidance on building confidence in managing their child’s specific learning and social difficulties.
4. Progress monitoring and feedback systems: Implementing simple tools for parents to track their child’s small achievements and receive regular positive feedback from teachers, reinforcing their sense of competence and effectiveness.
5. School-home collaboration platforms: Creating structured communication channels between teachers and parents that focus on collaborative problem-solving rather than merely reporting problems, thereby empowering parents as active partners in their child’s education.

Additionally, designing comprehensive intervention programs that simultaneously focus on increasing parental self-efficacy and modifying early maladaptive schemas could be particularly effective in enhancing the social interest of students with learning disorders. Utilising these findings in developing educational packages for parents and teachers, as well as informing educational policies, will be an important step towards improving the psychosocial outcomes of these students.

## 6 Conclusion

The overall conclusion indicated that parental self-efficacy affects the social interest of students with learning disorders both directly and indirectly. Parental maladaptive schemas, by creating cognitive and behavioural obstacles, limit the translation of self-efficacy into effective parenting behaviours. Therefore, to enhance the social interest of these students, adopting a multidimensional approach, including increasing parental self-efficacy and schema therapy-based interventions to modify early maladaptive beliefs, seems essential.

## Data Availability

The original contributions presented in the study are included in the article/supplementary material. Further inquiries can be directed to the corresponding author.
